# Acoustics of Emotional Prosody Produced by Prelingually Deaf Children With Cochlear Implants

**DOI:** 10.3389/fpsyg.2019.02190

**Published:** 2019-09-30

**Authors:** Monita Chatterjee, Aditya M. Kulkarni, Rizwan M. Siddiqui, Julie A. Christensen, Mohsen Hozan, Jenni L. Sis, Sara A. Damm

**Affiliations:** Auditory Prostheses and Perception Laboratory, Center for Hearing Research, Boys Town National Research Hospital, Omaha, NE, United States

**Keywords:** acoustics, emotion, vocal, production, speech, cochlear implants, children

## Abstract

**Purpose**: Cochlear implants (CIs) provide reasonable levels of speech recognition quietly, but voice pitch perception is severely impaired in CI users. The central question addressed here relates to how access to acoustic input pre-implantation influences vocal emotion production by individuals with CIs. The objective of this study was to compare acoustic characteristics of vocal emotions produced by prelingually deaf school-aged children with cochlear implants (CCIs) who were implanted at the age of 2 and had no usable hearing before implantation with those produced by children with normal hearing (CNH), adults with normal hearing (ANH), and postlingually deaf adults with cochlear implants (ACI) who developed with good access to acoustic information prior to losing their hearing and receiving a CI.

**Method**: A set of 20 sentences without lexically based emotional information was recorded by 13 CCI, 9 CNH, 9 ANH, and 10 ACI, each with a happy emotion and a sad emotion, without training or guidance. The sentences were analyzed for primary acoustic characteristics of the productions.

**Results:** Significant effects of Emotion were observed in all acoustic features analyzed (mean voice pitch, standard deviation of voice pitch, intensity, duration, and spectral centroid). ACI and ANH did not differ in any of the analyses. Of the four groups, CCI produced the smallest acoustic contrasts between the emotions in voice pitch and emotions in its standard deviation. Effects of developmental age (highly correlated with the duration of device experience) and age at implantation (moderately correlated with duration of device experience) were observed, and interactions with the children’s sex were also observed.

**Conclusion:** Although prelingually deaf CCI and postlingually deaf ACI are listening to similar degraded speech and show similar deficits in vocal emotion perception, these groups are distinct in their productions of contrastive vocal emotions. The results underscore the importance of access to acoustic hearing in early childhood for the production of speech prosody and also suggest the need for a greater role of speech therapy in this area.

## Introduction

Emotional communication is a key element of social development, social cognition, and emotional well-being. Studies have shown that in children and adults with cochlear implants (CIs), performance in vocal emotion recognition tasks predicts their self-perceived quality of life, but their general speech recognition does not (Schorr et al., 2009; Luo et al., 2018). This indicates that speech emotion communication is a critical area of deficit in CIs that needs to be addressed. Acoustic cues signaling vocal emotions in speech include voice pitch, timbre, intensity, and speaking rate (e.g., Banse and Scherer, 1996). Among these, voice pitch is a dominant cue. CIs do not represent voice pitch to the listener with adequate fidelity, but other cues to vocal emotions, such as intensity and duration cues, are retained in the electric input. These deficits in vocal pitch perception have been implicated in CI users’ poorer performance in pitch-dominant areas of speech perception such as prosody or lexical tones (Peng et al., 2004, 2008, 2017; Green et al., 2005; Chatterjee and Peng, 2008; See et al., 2013; Deroche et al., 2016; Jiam et al., 2017). The importance of voice pitch for spoken emotions is thought to account for the deficits observed in cochlear implant users’ ability to identify emotional prosody (Luo et al., 2007; Hopyan-Misakyan et al., 2009; Chatterjee et al., 2015; Paquette et al., 2018). The perceptual deficit observed in CI users in emotion identification suggests that on their own, these secondary cues are not sufficient to provide normal levels of accuracy in vocal emotion identification. Similar deficits have been observed in normally hearing listeners attending to CI-simulated speech (Luo et al., 2007; Chatterjee et al., 2015; Gilbers et al., 2015; Tinnemore et al., 2018).

Prelingually deaf children who received a CI (CCI) within the sensitive period (e.g., by 2 years of age) and are developing oral communication skills through the prosthesis provide a unique opportunity to investigate the impact of the perceptual deficits associated with electric hearing on the development of emotional prosody. This population also provides an important contrast to postlingually deaf adult CI users (ACI) who learned to hear and speak with good hearing in childhood before losing their hearing as teenagers or adults, in many cases in middle age or later years. ACI generally retain excellent speech production skills, despite listening through the distorted input of the CI. In a previous study comparing ACI and CCI in their vocal emotion perception, Chatterjee et al. (2015) noted that they were similar in both the mean and the range of performance. Notably, the stimuli used by Chatterjee et al. (2015) were highly recognizable by normally hearing listeners as they were produced in a child-directed manner, with exaggerated prosody. While few studies have reported deficits in prelingually deaf pediatric CI users’ productions of vocal emotions (Nakata et al., 2012; Van De Velde et al., 2019), they have focused on younger children (<10 years of age) and used perceptual ratings of the productions as the outcome measure. Little is known about the factors predicting the acoustic features of these productions as children develop into teenagers, and no studies have reported on a comparison between pre and postlingually deaf CI users. Here, we present acoustic analyses of emotional prosody [a set of 20 emotion-neutral sentences (i.e., without lexically based emotional information) read with “happy” and “sad” emotional prosody] produced by prelingually deaf school-aged children and postlingually deaf adults with CIs, alongside productions by typically developing normally hearing children and young normally hearing adults. We selected happy and sad emotions because these are well-contrasted acoustically (happy is spoken with a higher mean pitch, more fluctuating pitch, higher intensity, and faster than sad). These two emotions are also uncontroversial and relatively easy for school-aged children as young as 6 years old to know and be able to produce without an exemplar. Previous studies have used different methodologies, e.g., Nakata et al. (2012) asked children to imitate the vocal productions of an exemplar, while Van De Velde et al. (2019) asked children to produce a word depicted in a picture with an emotion simultaneously depicted in a picture. Imitative production provides information about vocal capabilities but not about how the participants would normally produce emotions. Van De Velde et al.’s (2019) method avoided imitation but may have imposed additional task complexity in the requirement to generate the word associated with the picture and the emotion associated with the picture, combine them conceptually, and produce the word with the correct emotion. In our task, we avoided imitation and kept the cognitive load to a minimum by asking children to read the list of sentences in a happy way and in a sad way. There was still the remaining task burden of having to combine the emotion with the sentence before producing it, but the participants did not have to generate the words themselves or figure out the emotion required for the production.

Among acoustic cues, we focused on mean voice pitch, variance of voice pitch, mean intensity, mean spectral centroid, and mean duration of each utterance. These cues were found to be important acoustic features of vocal emotions in previous studies (Banse and Scherer, 1996; Scherer, 2003). These cues have also been found to be useful in artificial manipulations of speech designed to represent different human emotions (e.g., Přibilová and Přibil, 2009). Based on pitch and spectral degradations in CIs, we expected the CI users (particularly CCI) to show deficits in the pitch and spectral centroid domains of their productions. We expected to observe smaller acoustic contrasts between “happy” and “sad” emotions in the productions of the CCI than in those by children with normal hearing (CNH) and adults with normal hearing (ANH), but we were interested in the specific acoustic cues that might show such reduced contrasts. We expected CNH and ANH to produce the emotions similarly. A key question of interest was how CCI and ACI would compare in their productions. Specifically, we asked if CCI and/or ACI would emphasize intensity or duration differences between the emotions to compensate for any deficits in the pitch domain. Previous studies have shown that adult and child CI users can trade primary acoustic cues for secondary cues such as duration and intensity in speech recognition, intonation recognition, and lexical tone recognition tasks (Peng et al., 2009, 2017; Winn et al., 2012). Luo et al. (2007) showed that removing intensity cues from the stimuli resulted in much poorer emotion recognition scores in their adult CI listeners, indicating that intensity cues are emphasized in vocal emotion recognition by postlingually deaf CI users. The extent to which this would influence their vocal emotion productions is not known, nor is it known whether prelingually deaf CCI would emphasize intensity cues in their productions. Among the CCI, we asked if earlier age at implantation or longer duration of experience with the device would change the acoustic characteristics of their productions. These questions center around the role of neuroplasticity within the more sensitive, early years of brain development and during the developmental period of auditory and language systems, which extends into the teenage years.

## Materials and Methods

### Participants

Participants were comprised of four groups of talkers. All talkers provided informed consent to be recorded, and procedures were approved by Boys Town National Research Hospital’s IRB protocol #11-24-XP. The four groups of talkers are as described below. Detailed information about the CI users who participated is shown in Table 1. The information in Table 1 was derived from a questionnaire filled out by participants or (in the case of child participants) by their parents/guardians. Written informed assent was obtained from all child participants, together with written informed parental consent to participate; written informed consent was obtained from all adult participants. Participants were compensated for travel time and for their listening time. In addition, children were offered a toy or a book of their choice after they completed their sessions.

**Table 1:** Information about CI participants.

	Age at testing (years)	Age of implantation (years)	Duration of CI use (years)	Bilateral implant (yes/no)	Sex	Manufacturer/device
**Prelingually deaf CCI group**						
Child CI participant						
CICH02	18.14	2	16.14	No	Male	Cochlear
CICH03	11.89	1.4	10.49	No	Female	Advanced Bionics
CICH13	7.72	0.83	6.89	Yes	Female	Advanced Bionics
CICH18	17.2	1.7	15.5	No	Female	Advanced Bionics
CICH19	7	0.9	6.1	No	Female	Advanced Bionics
CICH20	7.6	1.1	6.5	Yes	Male	Advanced Bionics
CICH22	12.62	1.86	10.76	Yes	Female	Advanced Bionics
CICH35	12.73	1	11.73	Yes	Male	Advanced Bionics
CICH36	16.27	1.5	14.77	Yes	Female	Med-El
CICH37	18.49	1.5	16.99	Yes	Female	Advanced Bionics
CICH38	7.9	1.25	6.65	Yes	Female	Cochlear
CICH39	16.61	1.17	15.44	Yes	Female	Advanced Bionics
CICH40	14.025	1.5	12.53	Yes	Male	Advanced Bionics
**Postlingually deaf ACI group**						
Adult CI participant						
C01	37	31	6	Yes	Female	Advanced Bionics
C03	67	55	12	No	Male	Advanced Bionics
C05	68	63	5	No	Female	Advanced Bionics
C06	75	55	20	No	Female	Advanced Bionics
C07	68	67	1	No	Female	Advanced Bionics
N5	53	50	3	No	Female	Cochlear
N6	51	44	7	Yes	Male	Cochlear
N7	57	51	6	No	Female	Cochlear
N15	61	59	2	No	Male	Cochlear
N16	27	25	2	Yes	Male	Cochlear

#### Children With Normal Hearing

Nine children with normal hearing participated. Their ages ranged between 6 and 18 years [mean age 12.5 years, standard deviation (SD) 4.4 years]. Five of the children were females, and four were males. All had normal hearing based on audiometric screening at criterion level of 20 dB HL or better between 250 and 8,000 Hz.

#### Children With Cochlear Implants

Thirteen children with cochlear implants participated. Their ages ranged between 7 and 18 years (mean age 12.93 years, SD 4.27 years). Four of the children were males, and nine were females. All of the CCI were prelingually deaf, implanted at the age of 2, and none had any usable hearing at birth. Their mean age at implantation was 1.36 years (SD 0.35 years), and their mean duration of device use was 11.57 years (SD 4.04 years).

#### Adults With Normal Hearing

Nine adults with normal hearing participated. Their ages ranged between 21 and 45 years. Six of the ANH were females; three were males. As with the CNH, normal hearing was confirmed based on audiometric screening at criterion level of 20 dB HL or better between 250 and 8,000 Hz.

#### Adults With Cochlear Implants

Ten postlingually deaf adults with cochlear implants participated. Their ages ranged between 27 and 75 years. Six of the ACI were females; four were males.

### Procedure

The list of materials used for this study is comprised of 20 simple sentences that had no overt semantic cues about emotion. These sentences are provided in Table 2 (identical to Table 2 in Damm et al., 2019, JSLHR). The sentences were simple enough that the youngest participants (as young as 6 years of age) could read them aloud easily. The protocol for the recordings was as follows: the participant was invited to sit in a soundproof booth at a distance of 12 inches from a recording microphone (AKG C 2000 B) and asked to read the 20 sentences in sequence, first in a happy way (three times) and then in a sad way (three times). They were provided with some initial practice runs and recordings that were initiated when they felt ready. No targeted training or feedback was provided; all feedbacks were encouraging and laudatory in nature. The signal from the microphone was routed through an external A/D converter (Edirol UA-25X) using Adobe Audition v. 3.0 or v. 6.0. Recordings were made at a sampling rate of 44,100 Hz and with 16-bit resolution. The recordings were high-pass filtered using a 75-Hz cut-off frequency. Of the three sets of recordings in each emotion provided by individual talkers, the second set was typically used for acoustic analyses. For instance in which the second recording of a particular sentence was noisy and included non-speech sounds (such as coughing or throat-clearing), the best sample of the other two recordings was selected. An order effect may be present in the data, as *happy* emotions were recorded prior to *sad.* The recordings took very little time overall, so it is unlikely that fatigue played a role. Based on experience, we noted that it was easier for the participants (particularly, the younger children) to begin the session with the *happy* productions and to continue recording in a particular emotion, rather than to switch from *happy* to *sad* during the recordings. Any order effect in the data would be expected to be present for all participants. The CI users who were bilaterally implanted were recorded with their earlier-implanted devices activated only.

**Table 2:** List of sentences.

*1. Time to go.*
*2. Here we are.*
*3. This is it.*
*4. This is mine.*
*5. The bus is here.*
*6. It’s my turn.*
*7. They are here.*
*8. Today is the day.*
*9. Time for a bath.*
*10. She is back.*
*11. It’s snowing again.*
*12. It’s Halloween.*
*13. Time for bed.*
*14. Time for lunch.*
*15. I see a dog.*
*16. I see a car.*
*17. I see a cat.*
*18. That is the book.*
*19. I saw a bug.*
*20. That is a big tree.*

#### Acoustic Analyses

Acoustic analyses were performed on the recordings using the Praat software package (Boersma, 2001; Boersma and Weenink, 2019). For the 40 recordings (20 sentences, 2 emotions each) provided by each participant, a Praat script was run to compute the mean pitch (F0, Hz), the F0 variation (standard deviation of F0), the mean intensity (dB), and the duration (sec) of each utterance. The default autocorrelation method in the Praat software program was used to estimate F0. Primary challenges in such analyses are encountered by researchers attempting to determine the onset and offset of the utterances in a consistent way and in setting parameters for pitch estimation appropriately for each utterance. The onset and offset times of each waveform were estimated using similar criteria by at least two of the co-authors so as to obtain consistent measures of duration. The pitch settings were established using the following steps: for each talker and emotion, a set of 4–5 recordings (from the total of 20) was pseudo randomly selected, and the pitch range, silence threshold, voicing threshold, octave cost, octave-jump cost, and voiced/unvoiced cost were set to appropriate levels, ensuring that the pitch contour was properly represented (e.g., avoiding octave jumps, discontinuities in the estimated pitch, or silences in regions of voiced speech). This was done more than once to ensure that the settings were indeed appropriate. Next, an automated Praat script was run on all the 20 recordings for that talker and emotion. The output was then analyzed for consistency (e.g., mean F0 values were compared across the recordings, and the ratio of maximum to minimum F0 for individual recordings was investigated). If these values appeared suspect for any of the recordings (e.g., if the ratio of maximum to minimum F0 values exceeded a value of 3.0 or if the estimated values were obviously different from other recordings by the same talker in the same emotion), they were individually checked again, modifications were made as needed to the settings, and the values were manually computed in Praat for those individual recordings. Two of the authors (RS and MC) were always involved in the final analyses. Some of the analyses of productions by the children had been previously conducted (by authors MC and JS) using a similar but not identical approach. Care was taken to compare these older analyses with the newer ones. When correlations between the two sets of data fell below 0.85, the analyses were again checked to ensure accuracy and modified again as needed.

Spectral centroid analyses were conducted in R using the seewave package (Sueur et al., 2008; Sueur, 2018). A window was first applied to discard the first 10% and last 10% of each waveform, with a bandpass filter with cut-off frequencies at [50, 4,000] Hz to narrow the range of the calculated centroid to speech content. Next, using the meanspec() function, the short-term Fourier transform (STDFT) of 50-ms long successive time segments (Hann-windowed with 50% overlap) of the waveform was computed and averaged across all segments to obtain the mean spectrum. Finally, using the specprop() function, the spectral centroid of each waveform was computed for its mean spectrum, based on the formula $C=\sum_{i=1}^{N}f_{i}\times a_{i}$, where *N* is the number of frequency bins (STDFT columns), *f_i_* is the center frequency of the *i*th bin, and *a_i_* is the relative amplitude. Both frequency and amplitude are linearly scaled in the centroid calculations.

#### Statistical Analyses

Statistical analyses and graphical renderings were conducted using R v. 3.3.2 (R Core Team, 2016). Plots were created using the *ggplot2* package within R (Wickham, 2016). Linear mixed effects models were constructed using the package *lme4* (Bates et al., 2015). A hierarchical approach was used to determine the best-fitting model, and the function *anova()* was used in the *car* package in R to compare models (Fox and Weisberg, 2011). Model residuals were visually inspected (using plots and histograms of residuals) to ensure normality. The *lmerTest* package (Kuznetsova et al., 2017) was used to obtain estimated model results and *t*-statistic-based significance levels for each parameter of interest. The *optimx* package (Nash and Varadhan, 2011) was used to promote model convergence in one instance.

## Results

### Group Differences

**Figure 1:**
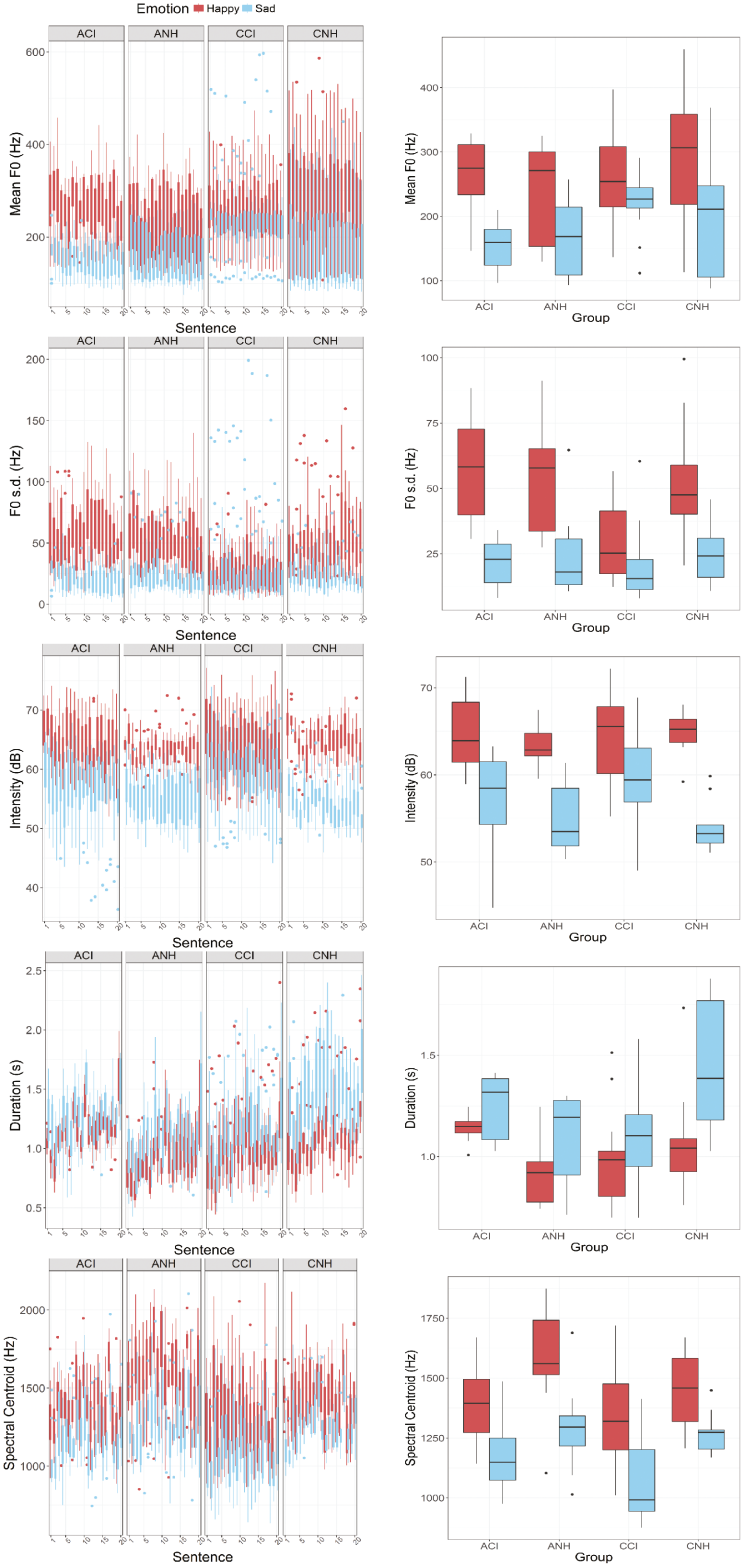
Group differences in acoustic features of emotional productions. **(Top to bottom – left panels)** These figures show boxplots of mean F0 (Hz), F0 s.d. (Hz), Intensity (dB), Duration (s), and Spectral Centroid (Hz) values estimated for each sentence (abscissa) recorded by the participants in each emotion (happy: red; sad: blue). Data from the four groups of participants are represented in the four panels (left to write: ACI, ANH, CCI, and CNH). **(Top to bottom – right panels)** These figures show boxplots of the mean values of these acoustic features computed across the 20 sentences recorded in each emotion by individual participants. The abscissa shows the four groups (ACI, ANH, CCI, and CNH). Happy and sad emotions are again shown in red and blue colors.

Figure 1 shows boxplots of the acoustic characteristics of happy and sad emotions produced by each of the four groups of participants. From top to bottom, the different rows show the mean F0, F0 variation [standard deviation of F0 (F0 s.d.)], mean intensity, duration, and spectral centroid of the sentences produced with the two emotions (red and blue). The boxplots in the left-hand panels show the distribution of values computed for each sentence (abscissa) across the participants. The boxplots in the right panel of each row show the mean values computed across the sentences recorded in each emotion.

LME analyses were conducted on these data to investigate effects of Group, Sentence, and Emotion and their interactions. In all cases, the LME model was constructed including Group, Sentence, and Emotion as fixed effects, subject-based random intercepts, and random slopes for the effect of sentence. The dependent variable in each case was the particular acoustic measure under consideration (mean F0, F0 s.d., Intensity, Duration, or Spectral Centroid). The effect of Sentence was included as a fixed effect because systematic differences for individual sentences were expected, based on differences between them in their phonetic and linguistic characteristics.

#### Mean F0

Results showed a significant interaction between Group and Emotion [*β* = −17.656 (SE = 3.879), *t*(1599.10) = −4.552, *p* < 0.0001] and a significant main effect of Emotion – i.e., higher mean F0 for happy than for sad productions [*β* = −45.014 (SE = 6.907), *t*(1599.1) = −6.517, *p* < 0.0001]. No other effects and no other interactions were observed. To follow-up on the interaction, we investigated the effect of Group for the happy and the sad productions separately. LME analyses on the happy productions with fixed effect of Group, by-subject random intercepts, and by-subject random slopes for the effect of individual sentences showed no effect of Group. A similar analysis on the sad productions did show a significant effect of Group [*β* = −25.96 (SE = 8.76), *t*(41) = −2.96, *p* = 0.005], explaining the interaction between Group and Emotion. A pairwise *t* test (Bonferroni correction) to investigate the effect of Group in the sad productions showed no significant differences between the ANH and ACIs’ mean F0 values (*p* = 0.32), but all other comparisons showed significant differences (*p* < 0.001 in all cases). Of note, the CCIs’ sad productions had the highest mean F0 of the four groups.

#### F0 Variation

The mean F0 and F0 s.d. values were significantly correlated in all groups. A linear multiple regression analysis confirmed that F0 s.d. was significantly predicted by mean F0 and also showed that there was an interaction with Group (i.e., different correlation coefficients for the different groups). Individual linear regression analyses within the four groups confirmed this observation: estimated coefficients for the ANH, ACI, and CCI groups were 0.266 (SE 0.01), 0.263 (SE 0.012), and 0.259 (SE 0.011), respectively, whereas the coefficient for the CNH group was only 0.162 (SE 0.009).

The LME analysis showed significant effects of Group [*β* = 9.307 (SE = 2.661), *t*(49.4) = 3.498, *p* = 0.001], as well as a significant interaction between Group and Emotion [*β* = −10.44 (SE = 1.569), *t*(1599) = −6.651, *p* < 0.0001]. No other effects or interactions were observed. Follow-up analyses showed that the effect of Group was significant for the happy emotion [*β* = 8.759 (SE = 2.774), *p* = 0.003], but no significant effect of Group was observed for the sad emotion. *Post hoc* pairwise *t* tests (Bonferroni corrections applied) comparing the F0 s.d. values obtained by the different groups for the happy emotion productions showed significant differences between the CCI group and ACI, ANH, and CNH groups (*p* < 0.0001 in all cases), but no significant differences between the ACI, ANH, and CNH groups. Thus, the CCI group’s productions for happy were more monotonous (smaller F0 s.d.) than all other groups.

#### Mean Intensity

Results showed a significant interaction between Group and Emotion [*β* = −0.757 (SE = 0.276), *t*(1558) = −2.738, *p* = 0.00625], a main effect of Emotion [*β* = −6.041 (SE = 0.492), *t*(1558) = −12.27, *p* < 0.0001], and a main effect of Sentence [*β* = −0.0773 (SE = 0.031), *t*(133.9) = 2.526, *p* = 0.0127].

The interaction between Group and Emotion was not clearly supported by follow-up analyses. When the data were separated out into happy and sad emotions, separate LME analyses with Group as a fixed effect, random subject-based intercepts, and random subject-based slopes for the effect of Sentence showed no significant effects of Group for either emotion. However, the estimated effect for Group [*β* = −1.1224 (SE = 0.686), *t*(41) = −1.635, *p* = 0.11] was larger for sad productions than for happy productions [*β* = −0.3044 (SE = 0.538), *t*(41) = 0.566, *p* = 0.574). This is likely explained by the somewhat lower intensity levels observed in CNH relative to other groups.

#### Duration

Results showed significant effects of Emotion [*β* = 0.2233 (SE = 0.0336), *t*(1599) = 6.8, *p* < 0.0001] and Sentence [*β* = 0.01244 (SE = 0.00194), *t*(1443) = 6.4, *p* < 0.0001] but no effects of Group and no two-way or three-way interactions.

#### Spectral Centroid

Results showed a significant effect of Emotion [*β* = −272.591 (SE = 30.093), *t*(1599) = −9.058, *p* < 0.0001] but no effect of Group or Sentence and no interactions.

### Acoustic Contrasts Between Happy and Sad Productions

The acoustic contrast between happy and sad productions was specifically investigated for each acoustic cue. For the mean F0, the contrast was defined as the ratio between the mean F0s for happy and sad productions. For the F0 s.d., the contrast was defined as the ratio between the standard deviations of F0 for happy and sad productions. For Intensity, the contrast was defined as the difference in dBs between mean intensities of happy and sad productions. For Duration, the contrast was defined as the ratio between the durations of happy and sad productions. For Spectral Centroid, the contrast was defined as the ratio between the spectral centroids of happy and sad productions. Ratios between the values for happy and sad productions were chosen over other measures (e.g., simple difference) for consistency with findings in the literature on auditory perception, which indicates that perceptual sensitivity to differences between sounds in specific acoustic dimensions are well modeled by a system that encodes the sensory input using a power law and/or logarithmic representation. LME analyses were conducted with Group and Sentence as fixed effects and by-subject random intercepts and Sentence as by-subject random slopes.

#### Mean F0 Contrasts

**Figure 2:**
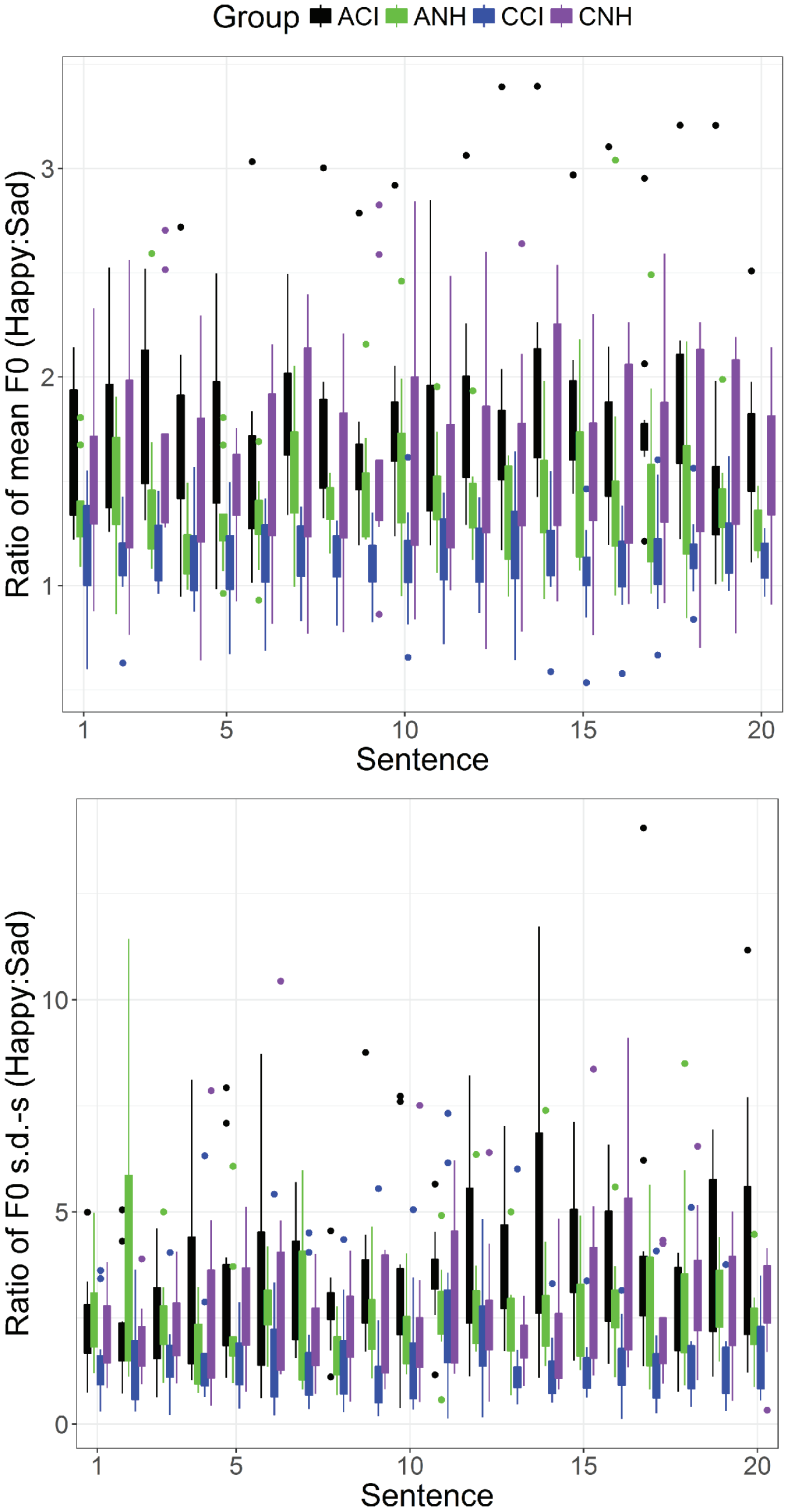
Boxplots of acoustic contrasts between happy and sad emotions for mean F0 **(upper)** and F0 s.d. **(lower)** for each sentence (abscissa) and for the four groups (see legend).

Results of the LME analysis showed a significant effect of Group [*β* = 0.115 (SE = 0.05), *t*(39.00) = 2.237, *p* = 0.031]. A pairwise *t* test with Bonferroni corrections showed significant differences between all Groups (*p* < 0.0001 in all cases). Figure 2 (upper) shows boxplots of the mean F0 contrast for the four groups and for each of the 20 sentences. The CCI group (blue) shows the smallest contrast of all four groups.

#### F0 Standard Deviation Contrasts

Results of the LME analysis showed a significant effect of Group [*β* = 0.35 (SE = 0.160), *t*(39.00) = 2.19, *p* = 0.0345]. A pairwise *t* test with Bonferroni correction showed significant differences between all groups (*p* < 0.0001 in all cases). Figure 2 (lower) shows boxplots of the F0 s.d. contrast for the four groups and for each of the 20 sentences. The CCI group shows the smallest contrast of all four groups.

#### Intensity Contrast

The results of the LME analysis showed no significant effects of Sentence or Group and no interactions.

#### Duration Contrast

Consistent with previous analyses, results of the LME analysis showed no effects of Group or Sentence and no interactions.

#### Spectral Centroid Contrast

Consistent with previous analyses, the LME analysis showed no effects of Group or Sentence and no interactions.

### Analyses of Results Obtained in Child Participants With Normal Hearing and Cochlear Implants

**Figure 3:**
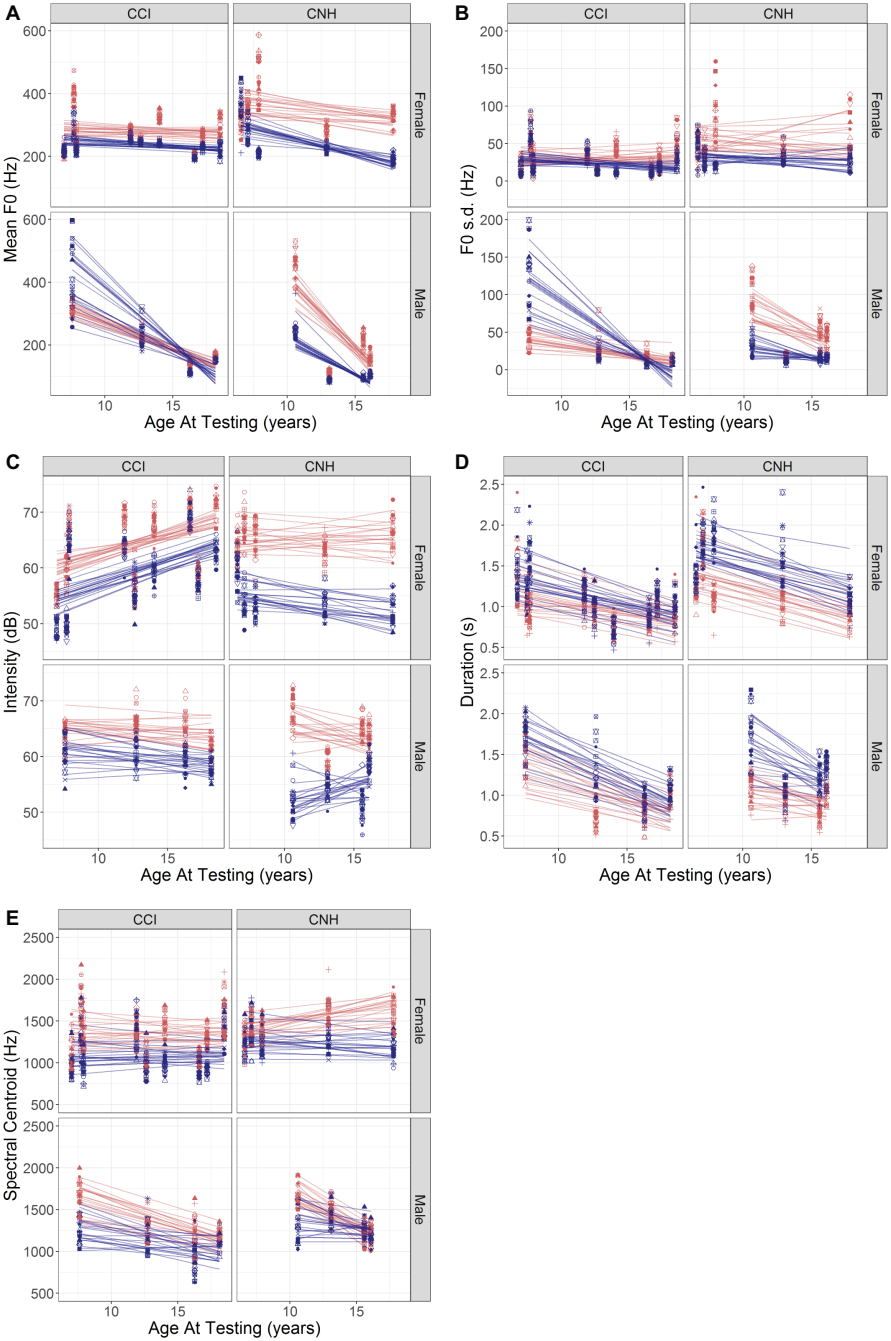
**(A–E)** Values of acoustic features (**A**: mean F0; **B**: F0 s.d.; **C**: Intensity; **D**: Duration; **E**: Spectral Centroid) of the happy (red) and sad (blue) emotions recorded by CNH and CCI, plotted against their age (abscissa). For each acoustic feature, left- and right-hand panels show results in CCI and CNH, respectively, and upper and lower plots show results in female and male participants, respectively. The differently shaped symbols and lines in each color represent individual sentences recorded in each emotion.

Initial analyses indicated different patterns for CNH and CCI and for female versus male children. Results obtained in NH and CI child participants were therefore analyzed separately for effects of Age and Sex on mean F0, F0 variation, Intensity, Duration, and Spectral Centroid. The data are plotted in Figure 3, which shows each acoustic cue as a function of Age, separated out by Sex and Group.

#### Acoustic Analyses of Productions by Children With Normal Hearing

##### Mean F0

An LME with fixed effects of Age and Sex and Sentence|Subject random intercepts/slopes showed significant effects of Sex [*β* = 407.03 (SE = 172.935), *t*(11.4) = 2.354, *p* = 0.0375], a significant interaction between Age and Sex [*β* = −37.29 (SE = 12.72), *t*(11.4) = −2.932, *p* = 0.0132], a significant interaction between Sex and Emotion [*β* = −242.569 (SE = 112.977), *t*(351) = −2.147, *p* = 0.0325], and a significant three-way interaction between Age, Sex, and Emotion [*β* = 19.348 (SE = 8.31), *t*(351) = 2.328, *p* = 0.0205]. The data are plotted in Figure 3A (right-hand panels). Consistent with expected differences in vocal development, the male children showed a larger decrease in F0 with age than female children did. The male children in this sample also showed a decreasing effect of emotion with age compared to the female children (hence, the three-way interaction).

##### F0 Variation

A parallel analysis to that described above with F0 s.d. as the dependent variable showed significant interactions between Age and Sex [*β* = −10.77 (SE = 3.33), *t*(13.6) = −3.022, *p* = 0.0094] and Sex and Emotion [*β* = −80.765 (SE = 39.08), *t*(342) = −2.067, *p* = 0.0395]. The pattern of results (Figure 3B, right-hand panels) is generally similar to that in Figure 3A for CNH, consistent with the correlation between the two variables. The separation between the emotions is somewhat smaller for male than for female participants in this sample, and there are age-related declines in the F0 s.d. in the male participants’ productions that are not observed in the female participants’ voices.

##### Intensity

An LME model with random slopes showed a significant effect of Emotion [*β* = 6.393 (SE = 2.364), *t*(351) = −2.704, *p* = 0.0072], a marginally significant two-way interaction between Age and Emotion [*β* = 0.3916 (SE = 0.209), *t*(351) = −1.873, *p* = 0.062], a significant two-way interaction between Sex and Sentence [*β* = 0.8233 (SE = 0.404), *t*(351) = 2.037, *p* = 0.0425], a three-way interaction among Sex, Emotion, and Sentence [*β* = −1.447 (SE = 0.572), *t*(351) = −2.531, *p* = 0.0118], a marginally significant three-way interaction among Age, Sex, and Sentence [*β* = −0.0559 (SE = 0.0297), *t*(347.827) = −1.879, *p* = 0.0611], and a four-way interaction among Age, Sex, Emotion, and Sentence [*β* = 0.099 (SE = 0.042), *t*(351) = 2.358, *p* = 0.0189]. The results are plotted in Figure 3C (right-hand panels). The separation between the emotions is clear, but for the male participants, the separation decreases somewhat of their age, more so than for female participants. The interaction with Sentence indicates that the pattern depends on the individual sentence.

##### Duration

An LME model with random slopes showed significant effects of Age [*β* = −0.051 (SE = 0.024), *t*(12.30) = −2.459, *p* = 0.0296] and Emotion [*β* = 0.420 (SE = 0.171), *t*(351)=2.456, *p* = 0.0145] with no other effects and no interactions. This is clearly apparent in Figure 3D (right-hand panels). The separation between the emotions remains consistent with age, across sentences, and for both sexes.

##### Spectral Centroid

An LME model with random slopes showed a significant effect of sex [*β* = 980.364 (SE = 359.645), *t*(14.30) = 2.726, *p* = 0.0162] and significant two-way interactions between Age and Sex [*β* = −84.471 (SE = 26.456), *t*(14.30) = −3.193, *p* = 0.0064], between Age and Emotion [*β* = −19.712 (SE = 9.686), *t*(352) = −2.035, *p* = 0.0426], and between Emotion and Sentence [*β* = 21.47 (SE = 9.145), *t*(352) = 2.348, *p* = 0.0195]. A three-way significant interaction among Sex, Emotion, and Sentence [*β* = −84.10 (SE = 26.493), *t*(352) = −3.174, *p* = 0.0016] and a four-way significant interaction among Age, Sex, Emotion, and Sentence [*β* = 5.471 (SE = 1.949), *t*(352) = 2.808, *p* = 0.0053] were also observed. The results are shown in Figure 3E (right-hand panels). It is apparent that the separation between the emotions decreases with age for the male participants, more so than for the female participants, and that the pattern varies across sentences.

#### Acoustic Analyses of Productions by Children With Cochlear Implants

##### Mean F0

Results obtained in child participants with CIs are plotted in the left-hand panels of Figure 3A. It is apparent that the separation between the emotions is smaller in the CI population than in the NH children (right-hand panels). A parallel analysis that conducted with CNH showed a significant interaction between Age and Sex [*β* = −12.971 (SE = 5.619), *t*(15.2) = −2.308, *p* = 0.0355]. This is clear in the steeper slope obtained with male children with CIs than in the female children with CIs in Figure 3A and parallels the findings with the CNH. A significant two-way interaction was observed between Sex and Emotion [*β* = 285.99 (SE = 42.159), *t*(507) = 6.784, *p* < 0.0001], and a three-way interaction among Age, Sex, and Emotion showed a further effect of Age on the Sex by Emotion interaction [*β* = −15.814 (SE = 3.018), *t*(507) = −5.24, *p* < 0.0001]. These interactions are likely explained by the female participants’ productions showing a consistent separation between happy and sad emotions with Age, while the male participants’ productions show little to no separation, which changes in direction with Age. A marginally significant three-way interaction among Sex, Emotion, and Sentence was also observed [*β* = −7.03 (SE = 3.519), *t*(507) = −1.998, *p* = 0.046], likely due to the greater dependence of the mean F0 on Sentence for sad emotions produced by male children relative to their happy emotions and also relative to their female counterparts (Figure 3A, left-hand panels).

##### F0 Variation

Results obtained in the child participants with CIs are plotted in the left-hand panels of Figure 3B. It is evident that the separations between the two emotions are smaller in the CCI than in their CNH counterparts (Figure 3B, right-hand panels). A similar analysis as described above with F0 s.d. as the dependent variable showed a significant two-way interaction between Sex and Emotion [*β* = 165.813 (SE = 18.646), *t*(507) = 8.893, *p* < 0.0001], three-way interactions among Age, Sex, and Emotion [*β* = −9.91 (SE = 1.334), *t*(507) = −7.425, *p* < 0.0001] and among Sex, Emotion, and Sentence [*β* = −3.991 (SE = 1.557), *t*(507) = −2.564, *p* = 0.0106], and a four-way interaction among Age, Sex, Emotion, and Sentence [*β* = 0.265 (SE = 0.111), *t*(507) = 2.378, *p* = 0.0178). The pattern of results is generally similar to those obtained with mean F0 (Figure 3B, left-hand panels).

##### Intensity

Results (plotted in the left-hand panels of Figure 3C) showed a marginally significant effect of Age [*β* = 0.781 (SE = 0.386), *t*(13.70) = 2.022, *p* = 0.063] and a significant effect of Emotion [*β* = −4.198 (SE = 1.692), *t*(494) = −2.481, *p* = 0.0134] and no other effects or interactions. It is apparent that the separation between the emotions is smaller in the children with CIs than in their counterparts with NH (Figure 3C, right-hand panels).

##### Duration

Results showed a significant negative effect of Age [*β* = −0.0368 (SE = 0.0137), *t*(18.1) = −2.662, *p* = 0.0158], indicating an overall faster speaking rate in older children, but no other effects or interactions. The effect of Age is similar to that observed in the children with NH.

##### Spectral Centroid

Results showed a significant effect of Emotion [*β* = −253.695 (SE = 121.996), *t*(508.2) = −2.08, *p* = 0.0381] but no other effects and no interactions. No obvious differences are apparent between the children with CIs and their NH counterparts.

#### Children With Cochlear Implants: Effects of Age at Implantation and Duration of Device Experience

The results obtained with CCI were separately analyzed for effects of Age at Implantation and Duration of Device Experience on individual acoustic cues to emotion. Age at implantation was significantly correlated with Duration of Device Experience (*r* = 0.63, *p* < 0.0001), so these variables were considered separately in the statistical analyses. Consistent with the Duration of Device Experience being highly correlated with Age (*r* = 0.99), the statistical analyses with Duration of Device Experience as a fixed effect produced almost identical results to those previously described with Age as the fixed effect and are not reported here in the interest of space. Results with Age at Implantation as the fixed effect of interest are described below.

LME analyses were conducted with Age at Implantation, Emotion, and Sentence as fixed effects, random intercepts by subject, and random slopes for the effect of Sentence.

##### Mean F0

**Figure 4:**
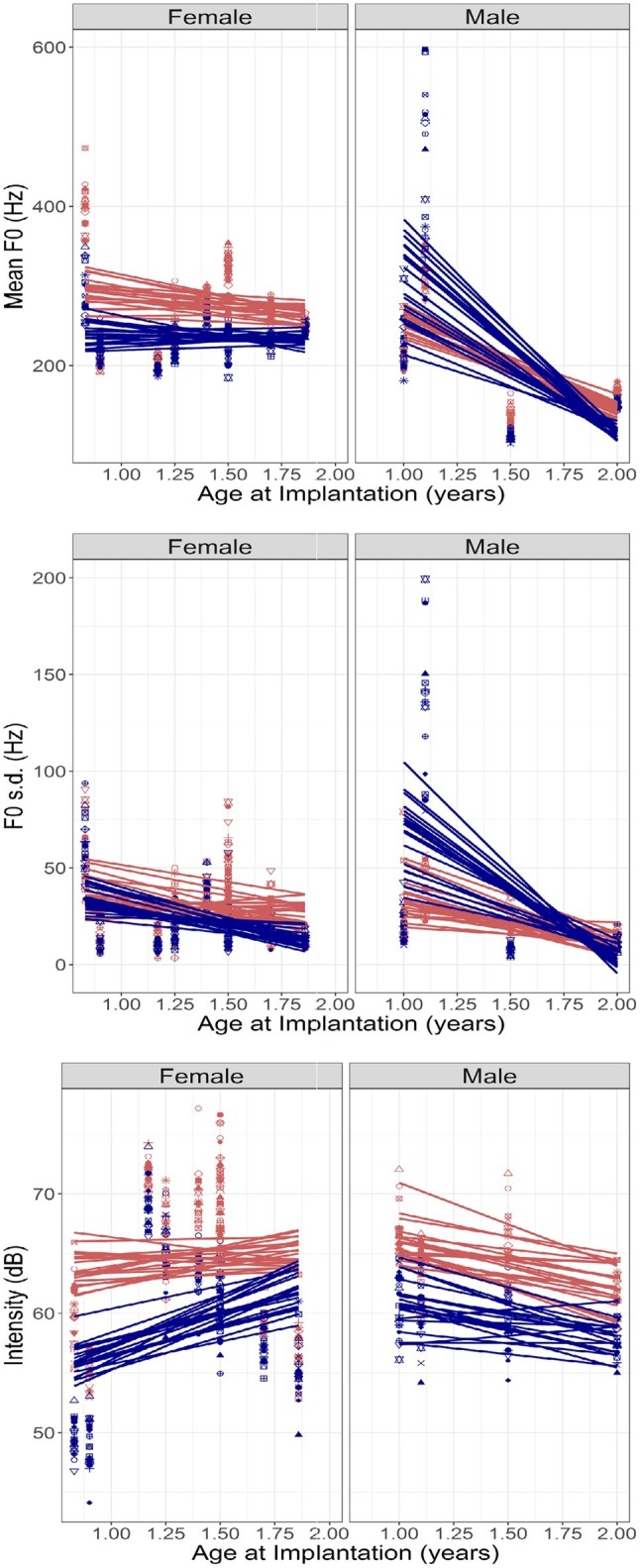
**(Top to bottom)** Mean F0, F0 s.d., and Intensity of productions by CCI, plotted against their age at implantation. Left- and right-hand panels show results in female and male participants, respectively. Red and blue symbols represent happy and sad emotions, respectively, and the differently shaped symbols and the lines represent individual sentences recorded in each emotion.

An LME analysis as described above with mean F0 as the dependent variable showed a significant effect of Emotion [*β* = −73.119 (SE = 30.5273), *t*(507) = −2.395, *p* = 0.017], a significant interaction between Emotion and Sex [*β* = 251.6315 (SE = 50.006), *t*(507) = 5.032, *p* < 0.0001], and a significant three-way interaction between Age at Implantation, Emotion, and Sex [*β* = −131.525 (SE = 35.046), *t*(507) = −3.753, *p* = 0.0002]. These interactions can be observed in Figure 4 (top panel), which plots the ratio of mean F0 values for happy and sad emotions against Age at Implantation. Left- and right-hand panels show data obtained in female and male children. The acoustic contrast for mean pitch is relatively unchanging for female children but increases for male children with increasing Age at Implantation. This likely simply reflects the developmental effects in the male children observed in Figure 3 (recall that Age at Implantation is correlated with age at testing).

##### F0 Variation

An LME analysis as described above with F0 s.d. as the dependent variable showed a significant interaction between Emotion and Sex [*β* = 94.7914 (SE = 24.711), *t*(507) = 3.836, *p* = 0.0001] and a three-way interaction between Age at Implantation, Emotion, and Sex [*β* = −46.195 (SE = 17.318), *t*(507) = −2.667, *p* = 0.0079]. Figure 4 (middle panel) shows the F0 s.d. ratio between happy and sad emotions plotted against Age at Implantation. The patterns are similar to those observed with mean F0 and also consistent with the effects of Age in Figure 3.

##### Intensity

An LME analysis as described above with Intensity as the dependent variable showed a significant effect of Emotion [*β* = −10.712 (SE = 2.169), *t*(494) = −4.938, *p* < 0.0001] and a significant interaction between Age at Implantation and Emotion [*β* = 4.715 (SE = 1.567), *t*(494) = 3.009, *p* = 0.0028]. A marginally significant three-way interaction between Age at Implantation, Emotion, and Sex was also observed [*β* = −4.71 (SE = 2.49), *t*(494) = −1.892, *p* = 0.0591]. Figure 4 (bottom panel) shows the intensity difference between happy and sad emotions plotted against Age at Implantation. The interaction between Age and Emotion appears to be determined by the female children who produce larger intensity differences (happy > sad) at earlier ages of implantation. This pattern of results is distinct from that observed in Figure 3 with Age. Note, however, that a nonlinear fit may have better captured the trends in this dataset, specifically, the elevated intensities observed at some ages at implantation: however, given the small sample size, we refrained from attempting such a fit to avoid problems with overfitting.

##### Duration

An LME analysis as described above with Duration as the dependent variable showed a marginally significant effect of Emotion [*β* = 0.3311 (SE = 0.18), *t*(507) = 1.842, *p* = 0.066], but no other effects or interactions reached significance.

##### Spectral Centroid

An LME analysis as described above with Spectral Centroid as the dependent variable showed a significant effect of Emotion [*β* = −380.227 (SE = 162.677), *t*(507.8) = −2.337, *p* = 0.0198] but no other effects and no interactions.

## Discussion

### Summary of the Results

Analysis of the mean F0, F0 variation (F0 s.d.), Intensity, Duration, and Spectral Centroid for the happy and sad emotions showed significant effects of Emotion on each of the cues measured. The mean F0, F0 s.d., mean Intensity, and Spectral Centroid were each higher for happy than for sad emotion productions, whereas Duration was shorter for happy than for sad. These basic findings are consistent with acoustic analyses reported in the literature in typical adult populations (e.g., Banse and Scherer, 1996). We were particularly interested in differences between the groups in the effect of the individual emotions. An interaction between Emotion and Group was observed for mean F0, F0 s.d., and Intensity but not for Duration or Spectral Centroid. The Group by Emotion interaction for Intensity was not well supported in *post hoc* analyses and was not reflected in the analysis of Intensity contrasts between the two emotions, which showed no significant effect of Group. Thus, the only reliably strong Emotion by Group interactions were those observed in F0 and F0 s.d. measures. The Emotion by Group interaction for mean F0 was explained by *post hoc* analyses indicating that mean F0 values were not significantly different for happy productions across the groups, but there was a significant difference between the pitch of sad productions between groups: while the adult NH and CI groups did not differ significantly, the CCI group produced a higher mean F0 for sad emotions than all others. *Post hoc* analyses on the F0 s.d. measures showed that the primary factor driving the Group by Emotion interaction was that the CCI group’s happy productions were the most monotonous of the four groups. Analyses of the acoustic contrasts between the two emotions further confirmed these findings.

The spectral centroid of an utterance provides information about the overall shape of the spectrum and is expected to be reflective of the phonetic content of the utterance, but it also provides information about emotion. Specifically, the relative energy in the lower and higher portions of the spectrum changes with emotion. As an example, Banse and Scherer (1996) showed that the decrease in energy at frequencies higher than 1,000 Hz is one of the important acoustic cues for emotion. These differences are reasonably well captured in the spectral centroid measure. For instance, positive emotions tend to be associated with more energy in the higher frequencies (higher spectral centroid), while negative or unpleasant emotions are associated with more energy at lower frequencies (lower spectral centroid). The present results suggest that all four groups showed similar changes in spectral centroid between happy and sad productions.

Consistent with the fact that the duration cue is well represented in CI processing, all four groups showed similar changes in duration with emotion, reflecting the expected faster speaking rate for happy emotion and a slower speaking rate for sad emotion. The Intensity cue is also represented in CIs, although the limited dynamic range and the effects of the automatic gain control do distort intensity-domain information, and this is consistent with the results showing that, similar to the other groups, the CCI also produced louder speech for happy than for sad emotions.

Taken together, the analyses of the group data indicate that the CCI produce happy and sad emotions with normal-range distinctions in duration, intensity, and spectral shape. The deficit appears to be focused on F0 (voice pitch)-related parameters in this dataset. Specifically, CCI produce smaller contrasts in mean F0 and in F0 variation than other groups. The reduced production of F0 contrasts is consistent with a degraded perception of voice pitch through CIs. The reduced F0 s.d. for happy emotions in CCI suggests a more monotonous speaking style overall, which may impose difficulties in social communication by this population. These data also suggest that CCI do not exaggerate contrasts between the cues as they are more perceptually sensitive (e.g., duration, intensity) to distinguish emotions in their speech. However, it is possible that differences do exist between CCI and other groups in these parameters and that a study with a larger sample size might reveal such differences. Based on the present dataset, it appears that F0-related cues are more strongly and more consistently impacted in CCIs’ productions than other cues.

The analyses of the CNH and CCIs’ productions were conducted separately to investigate developmental effects and effects of sex. Results in the CNH group showed interactions among Age, Sex, and Emotion, with the male children’s mean F0 decreasing more than the female children’s as they reached their upper teenage years. With the deepening voices, visual inspection of the data further suggested that the older male children also produced smaller contrasts between happy and sad emotions than did their female peers.

The CCI’s productions showed similar effects of Age and Sex, although the acoustic contrasts were clearly smaller for the CCIs’ productions than for the CNHs’ productions. Male CCI showed a deepening voice pitch with increasing age, while female CCI showed relatively small changes in voice pitch with age. The two younger male children with CIs showed a strong dispersion of mean F0 across sentences, particularly for sad productions, and higher mean F0 for sad than for happy productions for some of the sentences. The trend reversed in the older male children who showed the expected lower mean F0 for sad than for happy productions, but the separation remained small (Figure 3A). Note, however, that the limited sample size precludes the drawing of firm conclusions. Measures of F0 s.d. showed similar patterns. Intensity, Duration, and Spectral Centroid did not show any interactions between Age and Emotion in the CCI.

Analyses of effects of Age at Implantation and Duration of Device Experience were conducted separately because these two variables were correlated with one another. Duration of Device experience was highly correlated with Age, and the patterns of findings were virtually identical. Effects of mean F0 and F0 s.d. showed similar patterns with increasing Age at Implantation as those observed with Age and with Duration of Device Experience. The correlations between these variables preclude clear inferences regarding the underlying mechanisms. It is likely that the deepening mean F0 with Age at Implantation in male CCI is simply a reflection of developmental changes with Age.

The analysis of Intensity showed a different effect of Age at Implantation than did Age, and therefore, this effect is more likely to be unique to Age at Implantation. There was a significant two-way interaction between Age at Implantation and Emotion modified by Sex in a further three-way interaction. Visual inspection of Figure 4 (lower panels) suggests that the interaction was due to a greater separation of the emotions in earlier-implanted children than in later-implanted children, an effect that is stronger in female than in the male children in the present sample.

### Comparison Between Children With Cochlear Implants’ and Adults With Cochlear Implants’ Production of Emotions

Although both CCI and ACI hear speech through the degradation of CI processing combined with electric stimulation, the present results indicate that the two groups produce vocal emotions very differently. While the ACIs’ productions showed clear separations between the emotions in all measures considered, the CCI showed significantly smaller acoustic contrasts in F0 and in F0 variation than all other groups. On the other hand, ACIs’ perceptions of vocal emotions have been shown to be comparable to CCIs’ productions, even with the exaggerated prosody of child-directed speech (Chatterjee et al., 2015). This suggests that perception and production of vocal emotions may be linked in CCI who learned to speak through electric hearing, but not in ACI who learned to speak through acoustic hearing. We conclude that access to acoustic information in the early developmental years is crucial for the development of vocal motor patterns. In the ACI, these patterns seem to have been retained despite years of listening to a highly degraded, abnormal speech input. The CCI, on the other hand, had no access to usable hearing prior to implantation, and this is reflected in their atypical patterns of emotional prosody. We note here that the CCI produced the words in the sentences with high accuracy (this was separately verified by asking normally hearing listeners to listen to the recordings and repeat back the words in the recorded productions without regard to emotion). The ACI also produced the words with high accuracy. It is possible that speech therapy in CCI focuses more on speech phonetics of words than on speech prosody and that a greater focus on prosody in general may be beneficial to CCI. We note that ACI in the United States do not receive more than minimal speech therapy after implantation.

### Links to Related Studies in the Literature

Similar to the present study, other studies of vocal emotion production by children with CIs have also focused on primary emotions such as happy and sad primarily because they are highly contrastive in multiple acoustical dimensions as well as in their conceptual meaning. The present study focused on acoustic analyses, while other studies have investigated the intelligibility of the emotional productions. Additionally, in the majority of other studies, the child participants were tasked to imitate the emotional productions of an exemplar, while in the present study, participants were not provided with any examples, training, or targeted feedback. A recent study (Van De Velde et al., 2019) did not use imitative productions, but their methodology was quite different, and as discussed in the section Introduction, the task was more complex. These differences notwithstanding the present findings of reduced acoustic contrasts between the emotions in CCI are consistent with the findings of previous studies showing impaired or less recognizable emotions produced by CCI. These findings are also consistent with previous findings of impaired production of question/statement contrasts and lexical tones by children with CIs. Studies of singing by children with CIs also show impairments, although music requires a far greater sense of pitch, and therefore, singing may be considered a far more difficult task than producing speech intonations. Our finding that Age at Implantation had modest effects on the productions whereas Age at Testing (highly correlated with Duration of Device Experience) had a stronger impact is consistent with the findings of Van De Velde et al. (2019), who also found improvements with increased hearing age in their cohort of children with CIs.

In a recent investigation (Damm et al., 2019), the identifiability of these identical recordings made by the same participants was measured by asking normally hearing child and adult listeners to indicate whether each recording sounded happy or sad. In contrast to the normally hearing talkers and the postlingually deaf adult talkers, the CCI group’s recordings showed deficits in how well their recorded emotions were identified. In that study, Age at Implantation was found to be a significant predictor, with the earlier-implanted CCIs’ emotions being significantly better identified than the later-implanted CCIs’ productions. The group results are consistent between the two studies (i.e., the CCI in the present study produced smaller acoustic contrasts than other groups, and their emotions were also more poorly identified than other groups in the Damm et al. study). However, a larger dataset would be needed to establish direct relationships between acoustic features in individual talkers’ emotion productions and how well they can be identified by listeners.

### Limitations, Strengths, and Clinical Implications of the Present Study

The present study suffers from several limitations. First, the limited sample size leads us to treat these findings with caution. Thus, it is possible that a larger sample size might reveal differences in acoustic features such as intensity, duration, or spectral centroid, which are significant but cannot be captured with a small dataset. Second, the information about Age at Implantation was obtained from parents or guardians of the child participants and could not be verified independently. Third, perceptual data on this cohort of CCI’s emotion recognition abilities were not obtained, nor were data on their general or social cognition or other linguistic abilities. Fourth, the correlations between specific variables of interest (such as age at implantation and duration of device experience) precluded investigations of their combined effects. This, however, is a problem that is inherent to CI studies and not easily remedied in experimental design. Further, information about access/use of speech therapy in the CCI was not obtained. Finally, the method used to elicit the emotions had some limitations in that spontaneous expression of emotions was not achieved. There may well be differences between the emotions recorded using brief sentences in the laboratory and natural emotions communicated by the participants in their everyday life. Differences in the prosody of read or scripted speech as opposed to spontaneous speech have been reported in the literature (Laan, 1997). Although Damm et al. (2019) found that these methods evoked highly identifiable emotions in the CNH, ANH, and ACI groups, the differences between laboratory-recorded and naturally spoken emotional speech may further modify the group differences observed here. These limitations should be addressed in future studies.

Despite these limitations, the present results represent the first attempt to compare emotional productions by prelingually deaf children with CIs with postlingually deaf adult CI users, alongside normally hearing peers. One strength of the design was the careful selection of CCI who – with the caveat that the information was based on self- and parent-report and could not be independently verified – had no prior usable hearing at birth to more clearly separate them from postlingually deaf ACI who had good hearing in their early years. The findings suggest a key role of access to acoustic information during development for the production of prosodic cues. They also shed new light on specific sources of the impairment in emotional productions that could help develop improved speech therapy tools for children with CIs. For instance, the data suggest that the CCIs’ small acoustic contrast between mean F0 for happy and sad emotions in the present study was driven by an insufficiently low mean F0 for sad emotions compared to other populations. Additionally, CCIs’ small acoustic contrast between F0 variations for happy and sad emotions was driven by an overly monotonous production of happy emotions compared to other populations. These impairments may be addressed in targeted speech therapy. Finally, although the sample size was small, the findings suggest the possibility of differences between male and female children in their productions of vocal emotions and speech intonations. Specifically, the results suggest that male children with CIs may encounter difficulties adjusting to their changing vocal pitch with increasing age. This is an aspect that needs further investigation with larger sample sizes.

The emotions selected for the present study were chosen for their high acoustic and conceptual contrast. We speak with a higher pitch, with more pitch modulations, louder and faster when we communicate in a happy way. By contrast, we speak with a lower pitch, more monotonously, softer and slower when we communicate in a sad way. The vocal tract changes in a contrastive way between these emotions as well. The deficits observed in the CCI in the present study with these highly contrastive emotions may underestimate the true nature of the deficit when more subtle emotions are to be communicated through prosodic cues in speech. A study investigating how well these participants’ recordings were heard as happy or sad by normally hearing listeners (Damm et al., 2019) showed strong variability among the CCI talkers. Although some were very well understood, others’ emotions were mislabeled more frequently. Overall, the CCIs’ productions were less correctly identified than the ACIs’, the CNHs’, and the ANHs’ productions. On the other hand, the CCIs’ productions of the words in the sentences were highly recognizable. It is worth noting that present-day clinical protocols are designed with a focus on word and sentence recognition, with little to no emphasis on speech prosody. These findings, and others in the current literature, underscore a crucial need to address vocal pitch and emotion communication in the pediatric CI population in both the realms of scientific research and clinical intervention. The positive findings with ACI indicate that the presence of acoustic hearing (particularly at low frequencies) at birth and during development provides a supportive role in vocal emotion production, which is retained long after that hearing is lost. This result suggests a benefit to retaining any residual acoustic hearing in CCI alongside cochlear implantation, at least in the area of the production of emotional (and likely other forms) of speech prosody.

## Data Availability Statement

The datasets generated for this study are available on request to the corresponding author.

## Ethics Statement

The studies involving human participants were reviewed and approved by Boys Town Institutional Review Board. Written informed consent to participate in this study was provided by the participants’ legal guardian/next of kin.

## Author Contributions

MC conceptualized the study, led the design and set-up, led the acoustic analyses, analyzed the data, and wrote the manuscript. AK contributed to the study design and set-up, led the data collection, and processing. RS conducted acoustic analyses and helped with manuscript writing. JC contributed to study design and data collection and conducted preliminary acoustic analyses. MH contributed to study design and acoustic analyses. JS contributed to study design, data collection, and acoustic analyses. SD contributed to study design, data collection, and acoustic analyses.

### Conflict of Interest

The authors declare that the research was conducted in the absence of any commercial or financial relationships that could be construed as a potential conflict of interest.
